# Long non-coding RNA MALAT1 regulates hyperglycaemia induced inflammatory process in the endothelial cells

**DOI:** 10.1111/jcmm.12576

**Published:** 2015-03-19

**Authors:** Prasanth Puthanveetil, Shali Chen, Biao Feng, Anirudh Gautam, Subrata Chakrabarti

**Affiliations:** Department of Pathology, Schulich School of Medicine and Dentistry, Western UniversityLondon, Ontario, Canada

**Keywords:** endothelial cells, MALAT1, IL6, TNFα, kidney, diabetes, glucose

## Abstract

To examine whether the long non-coding RNA (lncRNA) metastasis associated lung adenocarcinoma transcript 1 (MALAT1) is altered in the endothelial cells in response to glucose and the significance of such alteration. We incubated human umbilical vein endothelial cells with media containing various glucose levels. We found an increase in MALAT1 expression peaking after 12 hrs of incubation in high glucose. This increase was associated with parallel increase in serum amyloid antigen 3 (SAA3), an inflammatory ligand and target of MALAT1 and was further accompanied by increase in mRNAs and proteins of inflammatory mediators, tumour necrosis factor alpha (TNF-α) and interleukin 6 (IL-6). Renal tissue from the diabetic animals showed similar changes. Such cellular alterations were prevented following MALAT1 specific siRNA transfection. Results of this study indicate that LncRNA MALAT1 regulates glucose-induced up-regulation of inflammatory mediators IL-6 and TNF-α through activation of SAA3. Identification of such novel mechanism may lead to the development of RNA-based therapeutics targeting MALAT1 for diabetes-induced micro and macro vascular complications.

## Introduction

Diabetes is a surging epidemic not only in North America but also worldwide. Among the diabetes-induced complications, retinopathy, nephropathy, cardiomyopathy, neuropathy and atherosclerosis, causing stroke and myocardial infractions are considered to be the major causes of morbidity and mortality in the diabetic population 1–3. The macro and micro vascular affections are key features of such complications. Endothelium is a major component of the vascular bed and one of the key cell type affected by glucose 1–3. As endothelium forms a protective barrier for all major organs in the body, maintaining endothelial homeostasis is essential for physiological organ function 1. Under conditions of nutrient excess, such as diabetes, endothelial insult leads to overproduction of specific cytokines causing an inflammation-like condition. Hence, understanding the molecular mechanisms causing endothelial inflammation would help prevent endothelial as well as end organ damage in diabetes 1,2. Several molecules have been identified as the major initiators and mediators of inflammation 1,3. Here, we reveal role for metastasis associated lung adenocarcinoma transcript1 (MALAT1), a 6.5 Kb nuclear residing long non-protein coding RNA, in hyperglycaemia induced endothelial damage. MALAT1 was originally shown to control of tumour metastasis and cancer cell survival 4. MALAT1 executes such functions by its ability to inhibit tumour cell death through induction of proteins like Bax, Bcl-2 and P53 5,6. These molecules are involved in cell cycle progression, vascularization and angiogenesis. However, specific mechanism(s) through which this non-protein coding RNA mediates downstream effects remains unidentified. Specifically, MALAT1's roles in noncancerous cells subjected to metabolic or nutrient stress haven't been investigated in depth.

Recently, MALAT1 has been identified as a regulator of inflammatory cytokine production in other systems 7. Study conducted by Yan *et al*. was first to show MALAT1 alteration in the retina in diabetes 8. Here, we investigated whether MALAT1 regulates inflammatory pathways involving inflammatory cytokines in diabetes.

Along with interleukin 6 (IL-6) and tumour necrosis factor alpha (TNF-α), we investigated mechanisms by which MALAT1 may regulate these cytokines through serum amyloid antigen (SAA). We further expanded these studies *in vivo*. Although both in type 1 and in type 2 diabetes, production of inflammatory cytokines such as SAA, IL-6 and TNF-α have been shown to play important roles, no studies have previously been performed exploring the transcriptional mechanisms, specifically with respect to that of the role of long non-coding RNAs (lncRNA), in glucose-induced endothelial damage 3,9. In this study, we have tried to identify whether in endothelial cells (*i*) glucose alters lncRNA, MALAT1, (*ii*) whether such alteration has functional significance, *i.e*. associated with regulation of downstream inflammatory mediators and (*iii*) whether changing MALAT1 expression corrects glucose-induced inflammatory cascades.

## Materials and methods

### Cell culture and reagents

Human umbilical vein endothelial cells (HUVECs) were obtained from Lonza (Canada Missisauga, ON) and were cultured in endothelial basal medium – 2. This medium was supplemented with the required growth factors (Lonza –growth factor Kit) and 5% foetal bovine serum. Following overnight serum starvation cells were incubated with 5 mM d-glucose (NG) or 25 mM d-Glucose (HG) or 25 mM l-Glucose (LG, osmotic control) or with other reagents as specified.

### Animal studies

All animal studies, were approved by the Western University Council on Animal Care Committee. The experiments were performed in accordance to The Guide for the Care and Use of Laboratory Animals (NIH guidelines, revised in 1996). Animals were made diabetic using Streptozotocin dissolved in citrate buffer maintained at a low acidic pH (70 mg/kg of mice, IP injection X3 on alternate days), non-diabetic (Control) animals were given the Citrate buffer alone. After the third dose of injection, hyperglycaemia was monitored using Freestyle Freedom Lite blood glucose monitoring system (Abbott Diabetes Care, Saint-Laurent, QC, Canada). Diabetic animals were maintained in hyperglycaemic state for 2 months on a standard rodent diet with water *ad libitum*. The animals were monitored regularly for blood glucose and body weight. At the end of 2 months, the animals were killed, kidneys were isolated and frozen for future use.

### Real time quantitative PCR

RNA was extracted using TRizol™ reagent (Invitrogen, burlington, ON, Canada). In brief, following Trizol treatment, chloroform was added to the cell lysate and the samples were centrifuged at 12,000 × g for 15 min. This was followed by subsequent treatment with isopropanol and wash with ethanol with centrifugation. Collected pellets were treated with RNAse free water. Following concentration determination by UV spectrophotometry, 2 μg of total RNA was used to make cDNA using reverse transcription kit from Applied Biosystems Inc, USA. The levels of target gene and house-keeping gene (18SRNA, Grand Island a widely used stable internal reference gene) were measured using a SYBR green dye in a light cycler (Roche Diagnostics, PQ). The primer sequences for human MALAT1 are forward primer Laval – CTTAAGCGCAGCGCCATTTT, reverse primer CCTCCAAACCCCAAGACCAA, human SAA3 – forward primer CTGGGTCACTGCTCCTCTTC, reverse primer ACTAGCACCTTTGCCCAGTG. Predesigned human TNF-α [Quantitech primer HS_TNF_3_SG (Cat no-QT01079561)] and human IL-6 [Quantitech primer HS_IL-6_1_SG (Cat no-QT00083720)] were obtained from Qiagen (Toronto, ON, Canada).

### ELISA assay

Following isolation of proteins from the endothelial cells using radioimmunoprecipitation assay buffer, protein concentrations were measured using BCA protein assay kit (Pierce, Rockford, IL, USA). Equal concentrations and amounts of total protein lysates from treatment groups were used each For ELISA. Human SAA 3 (cat no. CSB E11836h) kit was obtained from Cusabio-Biotech Co. Wuhan, Hubei, China For human TNF-α and human IL-6, ELISA kits (cat no. DTA00C and D6050 respectively) from R&D Systems (Mineapolis, MN, USA) were used. Following the treatments and incubation procedures as suggested by the vendors, the samples were read at 450 nm and background corrected at 568 nm.

### SiRNA transfection

SiRNAs targeted against human MALAT1 (product ID, nos'. 272231, 272233) were obtained from Life Technologies. In brief, all experimental groups were replenished with serum, growth factor and antibiotics free medium overnight. The cells were then either treated with SiRNA for human MALAT1 or the scrambled SiRNA (product ID, AM4635; Life Technologies) in Lipofectamine® 2000 transfection reagent (Invitrogen) following protocols provided by the vendors. Following initial assessments, we found that although both are effective, no. 272231 was slightly more robust (although both reduced MALAT1 RNA ∽65%; no. 272231 reduced SAA3 mRNA expression ∽50%, compared to ∽62% by no. 272233). Hence, in the subsequent experiments no. 272231 was used as MALAT1siRNA. For SiRNA for SAA we used SAA SiRNA (h) which is non-specific but targets SAA1, SAA2 and SAA3 (Santa Cruz Biotechnology, USA). We also used a second siRNA (cat no. AM16708; Life Technologies Santa Cruz, CA). Following a similar strategy as above, we used siRNA from Santa Cruz for subsequent experiments. Following transfection the cells were exposed to various treatments and were used for RT-qPCR and ELISA as above.

### Intracellular ROS measurement

After the HUVEC cells have reached 75% confluence, they were subjected to transfection and were exposed to normal and high glucose conditions in a serum free medium. Following 12 hrs of high or low glucose incubation, the cells were exposed to 2′,7′-dichlorofluorescein diacetate in required concentrations for 20–30 min. The reactive oxygen species levels were read by the fluorescence, emitted by the cell permeable dye converted to 2′,7′-dichlorofluorescein after being reacted with intracellular reactive oxygen species. The excitation and emission wavelengths were 492 and 521 nm respectively.

### Chemicals and reagents

All chemicals, unless otherwise specified, were obtained from Sigma Chemicals, Oakville, ON Canada.

### Statistical analysis

All experiments were performed in triplicate at least 3–5 times. The data are expressed as means ± S.E.M. Significance were determined using anova and/or Student's *t*-test with *post hoc* analysis using Graph Pad Prism software (La Jolla, CA, USA). The differences between groups were considered significant at the level of *P* < 0.05.

## Results

### Glucose stimulates early induction of MALAT1 in endothelial cells

**Figure 1:**
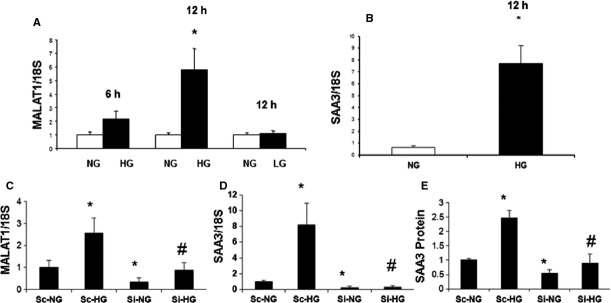
Hyperglycaemia induces SAA 3 gene expression and protein production in the endothelial cells through long non-coding RNA MALAT1. Endothelial cells were exposed to media containing normal (NG, 5 mM) and high d-glucose (HG, 25 mM) concentrations for various durations. RNA analyses by RT-PCR (mean ± S.E.) showed increase in (A) MALAT1 expression peaking at 12 hrs, but not when the cells were incubated with 25 mM of l-glucose (LG, osmotic control) in association with (B) increased SAA3 expression. (C) MALAT specific siRNA transfection (Si) caused significant reduction in basal- and glucose-induced MALAT1 up-regulation. Such knockdown of MALAT1 further normalized basal- and glucose-induced (D) SAA3 mRNA and (E) protein expression. [RNA data are expressed as a ratio to 18sRNA (mean ± S.E.), normalized to controls, *n* = 3 or more per groups, *: significantly different from corresponding NG, #: significantly different from corresponding HG, Sc: scrambled siRNA].

As endothelial cells are primary targets of glucose-induced damage, we investigated such changes in well-characterized HUVECs. Here, we found that 25 mM glucose (HG) up-regulates MALAT1 RNA expression compared to 5 mM glucose (NG). We found >2-fold increase MALAT1 expression levels after 6 hrs of incubation with HG, reaching to a peak of >5-fold increase after 12 hrs (Fig.1A). No such changes were seen with 25 mM l-glucose (osmotic control, Fig.1A).

### MALAT1 regulates inflammatory ligand expression in hyperglycaemia

Zhang *et al*. have shown that MALAT1 is dispensable for growth, development and survival under a normal physiological setting 4. In addition, they demonstrated that SAA3, a regulator of inflammatory cascade is a direct target of MALAT1. SAA3 is further diminished in the MALAT1 KO mice 4. In our study, incubation of endothelial cells in high glucose caused increase in SAA3 expression peaking at 12 hrs (Fig.1B), in association with increased MALAT1. We used siRNA to demonstrate a direct relationship both in low and high glucose conditions. Such siRNA transfection lead to >60% reduction in MALAT1 RNA in NG and lead to a significant drop in SAA3 expression (Fig.1C and D). Furthermore, such transfection of HUVECs in HG normalized glucose-induced increase in MALAT1 RNA and SAA3 mRNA expression (Fig.1C and D). We further examined SAA3 protein levels both in NG and HG conditions with or without SiRNA transfection. As expected, glucose-induced ∽2.5-fold increase in SAA3 protein, was normalized to control levels following siRNA knock down of MALAT1 in HG (Fig.1E). These data confirm that MALAT1 is a direct regulator of inflammatory ligand, SAA3, both under basal and under hyperglycaemic condition.

### MALAT1 inhibition attenuates the inflammatory response seen in hyperglycaemia

**Figure 2:**
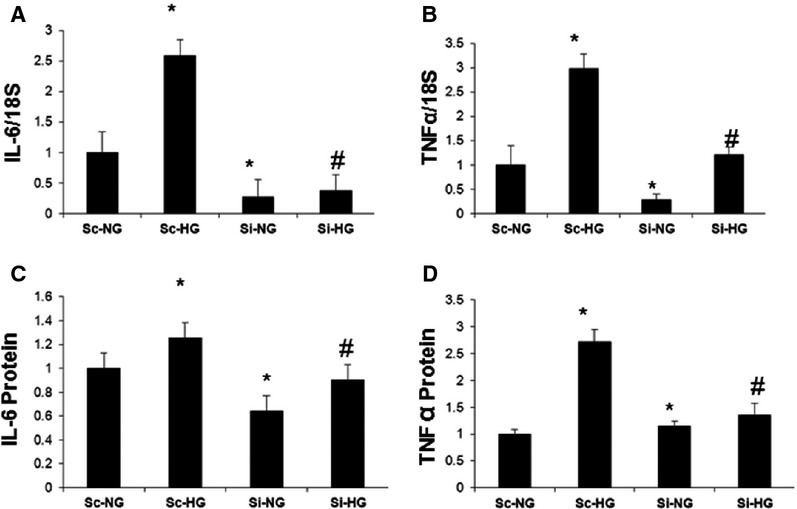
Hyperglycaemia regulates expression inflammatory genes IL-6 and TNF-α through MALAT1 in endothelial cells. (A) HUVEC were treated with normal (NG, 5 mM) and high glucose (HG, 25 mM) and were transfected with siRNA for MALAT1 (Si) and scrambled control (Sc). Such siRNA transfection reduced both basal- and glucose-induced up-regulation of (A) IL-6 and (B) TNF-α mRNAs, (C) IL-6 protein and (D) TNF-α protein [mRNA data are expressed as a ratio to 18sRNA and all data (mean ± S.E.) are normalized to controls, *: significantly different from corresponding NG, #: significantly different from corresponding high glucose groups, *n* = 3 or more per group].

Results from the previous studies have shown that SAA3 augments transcription of IL-6 and TNF-α with subsequent increase in the signalling of these classic inflammatory markers 9 in mice models. SAA 3 was considered a pseudogene in humans, till Larson *et al*., first showed that SAA 3 is regulated by prolactin and LPS in human mammary gland epithelial cells 10. Hence, we examined whether glucose-induced increased human SAA3 carries out similar downstream functions. In keeping with such notion, following 12 hrs of HG incubation, when the increase in MALAT1 and SAA3 reached the peak, there were augmented expression of mRNA and protein of IL-6 and TNF-α (Fig.2). Furthermore, siRNA mediated gene silencing of MALAT1 also showed reduction in IL-6 and TNF-α mRNA and protein levels confirming a direct regulatory relationship (Fig.2).

### MALAT1 induced inflammatory cascade is significantly regulated by SAA

**Figure 3:**
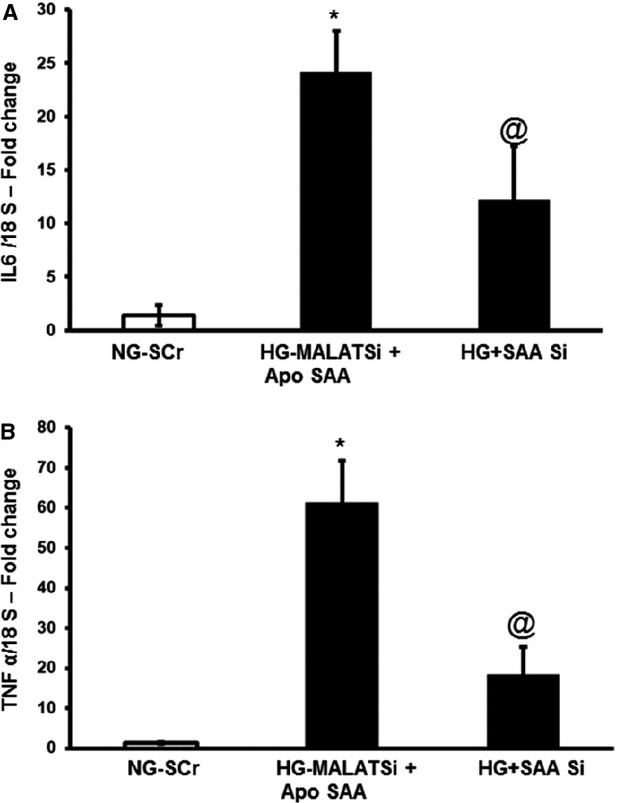
MALAT1 induced regulation of cytokine induction occurs mostly through SAA. HUVECs, treated with 5 mM glucose (NG) or 25 mM glucose (HG), were transfected with siRNA for MALAT1 and scrambled siRNA (SCr) used as control. Apo-SAA, a recombinant peptide, was used to stimulate SAA pathways and bring about downstream effects. (A) IL-6 and (B) TNF-α MRNAs were measured at 24 hrs time-point in the presence and absence of MALAT1 silencing. [mRNA data are expressed as a ratio to 18sRNA and all data (mean ± S.E.) are normalized to controls, *n* = 3 or more per group, *: significantly different from control and @: significantly different from Apo SAA treated groups with MALATSi, SAA Si: SAA3 siRNA].

To examine whether hyperglycaemia induced inflammatory markers induction by MALAT1 requires SAA, we tried silencing MALAT1 and added Apo–SAA, a recombinant protein. Addition of Apo SAA further aggravated the cytokine induction than high glucose alone at 12 hrs even in the presence of MALAT1 SiRNA. However, when SAA was silenced, we were able to bring down that drastic increase in IL-6 mRNA and TNF mRNA significantly (Fig.3) but could nt normalize to the control levels. These findings suggest that there are other pathways, in addition to the MALAT1-SAA axis may also be playing a role following hyperglycaemic stress.

### The early induction of MALAT1 in hyperglycaemia is not evident at later time-points and inflammatory cytokines follow similar pattern

**Figure 4:**
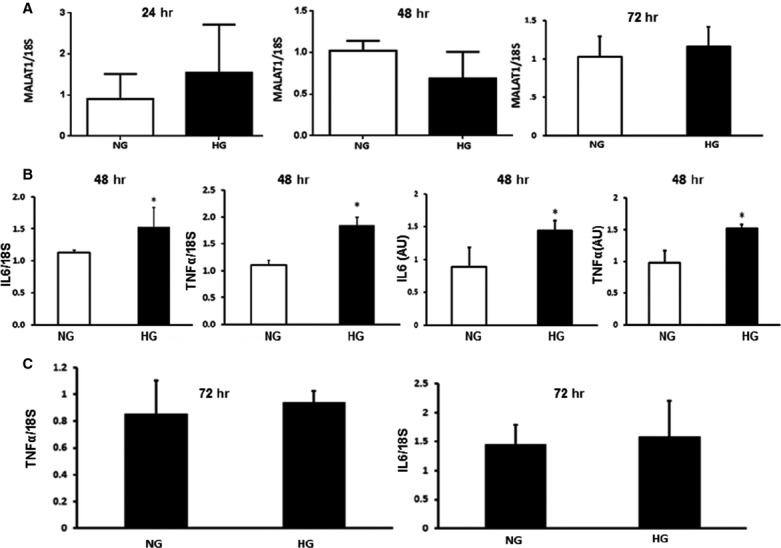
The effect of hyperglycaemia on MALAT1, IL-6 and TNF-α at later time-points. HUVECs incubated with 5 mM (NG) and 25 mM glucose (HG) showed (A) no significant alteration of MALAT1mRNA (measured by RT-PCR) expression after 24, 48 or 72 hrs. (B) IL-6 and TNF-α mRNA (measured by RT-PCR) and protein expressions (measured using ELISA) were increased following HG incubation at 48 hrs, but (C) returned to NG levels after 72 hrs of incubation [mRNA data are expressed as a ratio to 18sRNA and all data (mean ± S.E.) are normalized to controls, *n* = 3 or more per group, *: significantly different than control and *P* < 0.05, AU: arbitrary units of protein].

The early induction of MALAT1 RNA, seen following high glucose incubation of HUVECs was not apparent at 24, 48 or 72 hrs, compared to normal glucose (Fig.4A). It is possible that, either MALAT1 has a shorter half-life or their stability is influenced by hyperglycaemia at a longer time period. It is also possible that following initiation, other glucose-induced counter-regulatory mechanisms come into place. Interestingly, analysis of mRNA expressions of IL-6 and TNF-α at 48 hrs showed a sustained increase even when MALAT1 mRNA expression was normalized as shown in Figure4. However, in spite of an initial rise at 12 hrs time-point following hyperglycaemia (approximately 2.5-fold for both these transcripts), at 48 hrs the level of such up-regulations were lower (1.5- and 2-fold increase for IL-6 and TNF-α respectively). It could be possible that even though the stimulus by MALAT1 had subsided, the cytokines (IL-6 and TNF-α) could turn on their own gene and cause their mutual induction through a positive feedback loop. Even the protein expressions of IL-6 and TNF-α were high at this point, IL-6 protein was maintained at almost at the same level when we compared the fold change at 12 and 48 hrs protein levels. The early rise (12 hrs) in TNF-α protein (almost 2.5-fold) was reduced at 48 hrs (almost 1.5-fold) time-point. These findings may suggest that IL-6 and TNF-α may not be direct targets of MALAT1 and such regulations are mediated through downstream molecules, in this case SAA, thus prolonging the effects. However, exact cause(s) for this rise and fall of MALAT1 and downstream mechanisms remain to be identified and needs specific experiments, which are beyond the scope of this study.

### MALAT1 is increased in the kidneys of mice with diabetes

**Figure 5:**
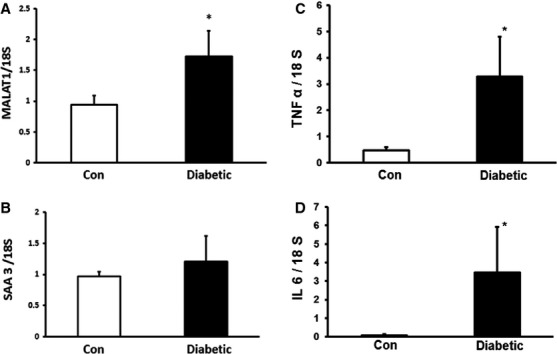
MALAT1 expression is up-regulated in the kidneys of diabetic mice. Streptozotocin induced diabetic mice maintained in the hyperglycaemic state for 2 months showed increased mRNA expression of both (A) MALAT1 and (B) SAA 3. Such increases were associated with (C) TNF-α and (D) IL-6 mRNA up-regulation (data are expressed as mean ± S.E., expressed as a ratio to 18sRNA, normalized to controls, *n* = 4–5/group, *: *P* < 0.05).

To examine whether such changes are of relevance *in vivo,* we analysed kidneys from streptozotocin induced diabetic mice and age-and sex-matched controls after 2 months of follow-up. As endothelial cells, lining the renal microvasculature are one of the first cell type exposed to the hyperglycaemic milieu, we thought that kidneys are good targets for analysing the inflammatory effects of hyperglycaemia. Diabetic mice were hyperglycaemic (diabetic mice 22.2 ± 3.6 mmol/l *versus* controls 6.8 ± 0.8 mmol/l, *P* < 0.001) polyuric and showed lower bodyweight (diabetic mice 23.5 ± 0.90 g *versus* controls 32.1 ± 3.6 g, *P* < 0.001). We saw a significant increase in MALAT1 RNA expression and a moderate increase in SAA3 mRNA expression (almost ∽20%) compared to the controls. Such changes were associated with increased IL-6 and TNF-α mRNA levels in diabetes (Fig.5).

### MALAT1 silencing dampens the early rise in oxidative stress

**Figure 6:**
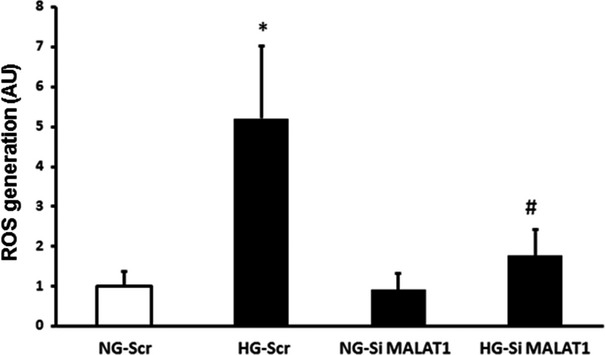
Effect of MALAT1 inhibition of endothelial ROS levels. In the endothelial cells, 25 mM glucose (HG) glucose-induced increased ROS production was prevented following MALAT1 SiRNA transfection (SiMALAT1) but not by scrambled siRNA (Scr) transfection (NG = 5 mM glucose, the data are mean ± S.E., *n* = 3 or more/group, *n* = 3 or more per groups, *: significantly different from NG-Scr, #: significantly different from HG-Scr, AU: arbitrary units).

We were interested in finding out whether early inhibition of MALAT1 (at 12 hrs) has functional significance in endothelium. Glucose-induced oxidative stress is a key alteration in the endothelial cells and is a mediator of most, if not all, other downstream effects 11. Hence, we measure oxidative stress as a functional indicator. We silenced MALAT1 and compared the ROS generation following hyperglycaemic stress at 12 hrs. We found a 4.5-fold increase in ROS production in high glucose condition, which was attenuated following MALAT1 silencing (Fig.6).

## Discussion

In this study we have shown that lncRNA MALAT1 is induced following short-term hyperglycaemia which turns on early inflammatory events in the endothelium through SAA3. Although the ability of hyperglycaemia to induce inflammatory markers has been previously studied, mechanistic role of a non-coding RNA to mediate such process has not been identified previously.

In a chronic disease like diabetes, low grade inflammatory changes associated with increased production of inflammatory mediators may be a key mechanism in the pathogenesis of chronic diabetic complications 12. Glucose-induced oxidative DNA damage may trigger increased expression of specific cytokines causing tissue damage. Here, we have identified a novel mechanism which may play a key role in such process at the transcriptional level. Identification of such process further opens up the possibilities of novel therapies targeting specific RNA molecules. It is well known that only 1.5% of the entire genome has protein-coding capacity 13,14. Non-protein coding RNAs are probably of importance and relevance in normal development and in various diseases through their capacity to regulate transcription and translation 13,14. Hence, they constitute potential drug targets capable of producing phenotypic changes. The specific roles of most of these lncRNAs remain mysterious although they are known to play regulatory roles in various, if not all, biological processes 13,14. MALAT1 (AKA NEAT2: noncoding nuclear-enriched abundant transcript 2, HCN, LINC000 47) is a large, infrequently spliced non-coding RNA, which is highly conserved. MALAT1 is expressed in the nucleus and was originally described to regulate metastasis and cell motility 4. MALAT1 is now known to be of importance in other disease processes 4. MALAT1 regulates cell survival through modulating p53 expression 5. With respect to other cytokines, MALAT1 has been shown to regulate TGFβ signalling by modulating activity of SMAD2/3 15. In addition, studies in MALAT1 knockout mice have shown SAA3 is regulated by MALAT1 and that SAA3 stimulates IL-6 and TNF-α production through NFκB and p38 mitogen activated protein kinase signalling 4,16. Till to date there has been no study revealing the signalling mechanisms associated with MALAT1 induction and how it could play a role in hyperglycaemic stress. Interestingly, we found early increase followed by a drop of MALAT1, which in turn positively up-regulate the inflammatory ligand like SAA3, ultimately augmenting production of inflammatory cytokines like IL-6 and TNF-α. However, this pattern is different than the cancer cells where such expression levels remain high 5,14. Further duration-dependent studies at various stages of diabetes using animal models (acute, short-term and long-term) would reveal whether this lncRNA, MALAT1 is a real culprit as an initiator of inflammation and oxidative stress. In keeping with such notion we have shown that MALAT1, through SAA3, also regulates glucose-induced inflammatory changes and oxidative stress. Such changes may influence endothelial stability. Endothelial homeostasis is essential for all organs and for macro and micro vessels. If we understand the switches involved in early endothelial inflammatory process, we may be able to retard the progress and consequences of such damage.

**Figure 7:**
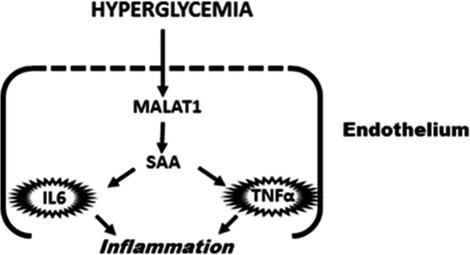
A schematic diagram depicting the mechanism of MALAT1 mediated regulation of inflammation in the endothelium in hyperglycaemia. Hyperglycaemia initiates the inflammatory response cascade through MALAT1 mediated up-regulation of serum amyloid antigen, thus stimulating the induction of inflammatory markers IL-6 and TNF-α. IL-6 and TNF-α can then maintain a sustained endothelial inflammation which sets the stage for cardiovascular diseases especially under nutrient stress.

In summary, in this study, we revealed a novel signalling nexus involving the lncRNA, MALAT1 and SAA3 which then turns on the inflammatory mediators in the endothelium as shown in the summary diagram (Fig.7). Understanding such mechanisms may eventually lead to novel RNA based therapeutic strategies for diabetic induced micro- and macro vascular complications. However, it is to be noted that the demonstrated role of MALAT1-SAA–Cytokine needs further evaluation in well-designed animal experiments with long-term diabetes. Such experiments may show whether MALAT1 may lend itself as a possible drug target in chronic diabetic complications.
